# Antiurolithiatic and antioxidant activity of *Mimusops elengi* on ethylene glycol-induced urolithiasis in rats

**DOI:** 10.4103/0253-7613.71925

**Published:** 2010-12

**Authors:** Purnima Ashok, Basavaraj C. Koti, A.H.M. Vishwanathswamy

**Affiliations:** Department of Pharmacology, K.L.E. University’s College of Pharmacy, II Block, Rajaji Nagar, Bangalore - 560 010, Karnataka, India; 1 Department of Pharmacology, K.L.E. University’s College of Pharmacy, Hubli, Karnataka, India

**Keywords:** Antiurolithiatic activity, BUN, creatinine, *Mimusops elengi*

## Abstract

**Objective:**

To evaluate the potential of *Mimusops elengi* in the treatment of renal calculi.

**Materials and Methods:**

Petroleum ether, chloroform, and alcohol extracts of *Mimusops elengi* bark were evaluated for antiurolithiatic and antioxidant activity in male albino Wistar rats. Ethylene glycol (0.75%) in drinking water was fed to all the groups (Groups II–IX) except normal control (Group I) for 28 days to induce urolithiasis for curative (CR) and preventive (PR) regimen. Groups IV, V, and VI served as CR, and groups VII, VIII, and IX as PR were treated with different extracts of *M. elengi* bark. Groups I, II, and III served as normal control, positive control (hyperurolithiatic), and standard (cystone 750 mg/kg), respectively. Oxalate, calcium, and phosphate were monitored in the urine and kidney. Serum BUN, creatinine, and uric acid were also recorded. *In vivo* antioxidant parameters such as lipid peroxidation (MDA), glutathione (GSH), superoxide dismutase (SOD), and catalase (CAT) were also monitored.

**Results:**

All the extracts of *M. elengi* were safe orally and exhibited no gross behavioral changes in the rats. In hypercalculi animals, the oxalate, calcium, and phosphate excretion grossly increased. However, the increased deposition of stone forming constituents in the kidneys of calculogenic rats were significantly (*P* < 0.001) lowered by curative and preventive treatment with alcohol extract (AlE) of *M. elengi*. It was also observed that alcoholic extract of *M. elengi* produced significant (*P* < 0.001) decrease in MDA, and increased GSH, SOD, and CAT. These results confirm that AlE of *M. elengi* possess potent antiurolithiatic activity.

**Conclusion:**

The results obtained suggest potential usefulness of the AlE of *M. elengi* bark as an antiurolithiatic agent.

## Introduction

Urolithiasis refers to the solid nonmetallic minerals in the urinary tract. Among the several types of kidney stones, the most common are calcium oxalate. The formation of these stones involves several physicochemical events, beginning with crystal nucleation, aggregation, and ending with retention within the urinary tract.[1] Several plants have been used to treat kidney stones including Phyllanthus niruri, Zea mays, Agropyron repens, and Herniaria hirsuta.[2–6] *Mimusops elengi* bark has been reported to possess hypotensive[7] and diuretic[8] activities. Phytochemical constituents such as alkaloids, tannin, saponius, taraxerone, ursolic acid, betulinic acid, mixture of triterpenoid saponium, etc. are present in the bark of *M. elengi*. On the basis of these facts and in continuation of our research work on *M. elengi*, we report herein the antiurolithiatic activity of various extracts of *M. elengi*.

## Materials and Methods

### 

#### Animals

Male Wistar albino rats weighing 150–200 g were used for the study, after obtaining approval from Institutional Animal Ethics Committee. Animals were acclimatized to laboratory conditions for 1 week before starting the experiment and had free access to water and standard rat feed. The animals were kept 12 h fasting prior to experiment.

#### Plant material

The bark of *M. elengi* was collected from Hubli, Karnataka, and was authenticated at the Department of Botany of H.S. Kothambri Science Institute, Hubli. A voucher specimen has been deposited at the herbarium for further reference.

#### Processing of plant material

The bark of *M. elengi* was shade dried at room temperature and was subjected to size reduction to get coarse powder of desired particle size. The powdered material was subjected to successive extraction in a Soxhlet apparatus using solvents petroleum ether (60–80°C), chloroform, and alcohol. Appearance of colorless solvent in the siphon tube was taken as the end point of extraction. The extracts were concentrated to ¾ of its original volume by distillation. The yield was 12.8% w/w, 3.5%w/w, and 18.2% w/w for petroleum ether, chloroform, and alcohol extract (AlE), respectively. The results of preliminary phytochemical investigation are shown in Table 1.

**Table 1:** Preliminary phytochemical analysis of *M. elengi* bark

*Chemical constituents*	*Petroleum ether extract*	*Chloroform extract*	*Alcohol extract*
Flavonoids	–	+	+
Glycosides	–	–	+
Sterols	+	+	–
Saponins	–	–	+
Phenolic compounds	–	–	+
Alkaloids	+	+	+

+ = Present; – = absent.

#### Materials and Methods

All the drugs, chemicals, and reagents were procured from S.D. Fine Chemicals (Mumbai, India). All the chemicals were of analytical grade. Analyzing kits were obtained from ERBA diagnostics, Daman, India.

#### Acute toxicity studies

Acute toxicity studies were carried out as per OECD guidelines (No: 423) using female Wistar mice by employing the Up and Down method prior to evaluating each of the extracts for antiurolithiatic activity.

#### Experimental design

Ethylene glycol-induced hyperoxaluria method[9] was used to assess the antiurolithiatic activity in albino Wistar rats. Animals were divided into nine groups containing six animals in each. Group I served as control and received regular rat food and drinking water ad libitum. Ethylene glycol (0.75%) in drinking water was fed to Groups II–IX for induction of renal calculi for 28 days. Group III received standard antiurolithiatic drug, cystone (750 mg/kg b.w.), from 15^th^ day till 28^th^ day.[10] Groups IV, V, and VI served as curative regimen (CR). Group IV received petroleum ether extract (PtE) (200 mg/kg b.w.) and Group V received chloroform extract (ChE) (200 mg/kg b.w.) from 15^th^ day till 28^th^ day, Group VI received alcohol extract (AlE) (200 mg/kg b.w.) and Group VII received (200 mg/kg b.w), VIII received (200 mg/kg b.w), and IX received AlE (200 mg/kg b.w.) from 1^st^ day till 28^th^ day and served as preventive regimen (PR). All extracts were given once daily p.o.

#### Assessment of Antiurolithiatic Activity

#### Collection and analysis of urine

Rats were kept separately in metabolic cages and urine samples of 24 h were collected on 28^th^ day. A drop of concentrated hydrochloric acid was added to the urine before being stored at 4°C. Urine samples were analyzed for calcium,[11] phosphate,[12] and oxalate[13] content.

#### Serum analysis

At the end of the experiment, blood samples were collected from the retro-orbital plexus under anesthetic conditions and analyzed for creatinine, urea nitrogen, and uric acid.[14]

#### Kidney homogenate analysis

A sample of 100 mg of the dried kidney was boiled in 10 mL of 1 N hydrochloric acid for 30 min and homogenized. The homogenate was centrifuged at 2000 × g for 10 min, and the supernatant was separated. The calcium, phosphate, and oxalate content in kidney homogenate were determined.

#### Enzyme assays

A portion of kidney was taken from all the groups, and a 30% w/v homogenate was prepared in 0.9% buffered KCl (pH 7.4) for the estimation of glutathione (GSH),[15] superoxide dismutase (SOD),[16] catalase (CAT),[17] and malondialdehyde (MDA).[18]

#### Statistical analysis

The results were expressed as the mean ± SEM and analyzed using one-way ANOVA followed by Dunnett’s multiple comparison tests. Data were computed for statistical analysis using Graph Pad Prism Software and *P* < 0.05 was considered to be statistically significant.

## Results

Acute toxicity studies observed that animals tolerated a maximum dose of 2000 mg/kg b.w. with no noticeable behavioral changes in all groups. Therefore, 1/10^th^ of the maximum tolerated dose 200 mg/kg b.w. was chosen for further studies.

Hyperoxaluria induced by chronic administration of 0.75% (v/v) ethylene glycol increased oxalate, calcium, and phosphate excretion [Table 2, Group II]. Alcohol extract of *M. elengi* bark significantly (*P* < 0.001) lowered the elevated levels of oxalate, calcium and phosphate in urine and kidney in CR and PR as compared to PtE and ChE in calculi-induced animals [Table 2, Group VI]. The deposition of the crystalline components in the renal tissue, namely oxalate, phosphate, and calcium, were increased in the stone forming rats (Group II). The AlE of *M. elengi* bark treatment significantly (*P* < 0.001) reduced the renal content of stone forming constituents in both regimens (Groups VI and IX). The serum uric acid, creatinine, and BUN were significantly increased in calculi-induced animals (Group II) as compared to control, while serum creatinine was elevated in Group II, indicating marked renal damage. However, AIE of *M. elengi* bark extract treatment in CR (Group VI) and PR (Group IX) significantly (*P* < 0.001) lowered the elevated serum levels of creatinine, uric acid, and BUN.

**Table 2:** Effects of various extracts of *M. elengi* bark on oxalate, calcium, phosphate in rats

*Parameter (unit)*	*Group I, control*	*Group II, calculi induced*	*Group III, cystone treated*	*Curative regimen*			*Preventive regimen*		
				*Group IV, PtE*	*Group V, ChE*	*Group VI, AlE*	*Group VII, PtE*	*Group VIII, ChE*	*Group IX, AlE*
Urine (mg/dL)									
Oxalate	0.28 ± 0.01	2.61 ± 0.17*a	0.46 ± 0.10*	2.28 ± 0.30	2.40 ± 0.20	1.30 ± 0.18*	2.20 ± 0.20	2.18 ± 0.40	0.84 ± 0.02*
Calcium	1.18 ± 0.03	3.85 ± 0.18*a	1.34 ± 0.10*	3.52 ± 0.36	3.38 ± 0.32	2.18 ± 0.19*	3.22 ± 0.30	3.20 ± 0.22	2.10 ± 0.14*
Phosphate	2.96 ± 0.04	6.82 ± 0.13*a	3.36 ± 0.03*	6.50 ± 0.28	6.56 ± 0.47	4.62 ± 0.12*	5.89 ± 0.29*	6.20 ± 0.30	3.31 ± 0.16*
Kidney (mg/g)									
Oxalate	1.36 ± 0.02	4.85 ± 0.11*a	2.15 ± 0.03*	4.12 ± 0.38	4.52 ± 0.18	3.18 ± 0.08*	4.25 ± 0.24	4.38 ± 0.26	2.28 ± 0.01*
Calcium	3.02 ± 0.03	4.94 ± 0.21*a	1.82 ± 0.27*	4.26 ± 0.32	4.30 ± 0.22	3.22 ± 0.11††	4.42 ± 0.38	4.32 ± 0.44	2.50 ± 0.28*
Phosphate	2.58 ± 0.04	3.88 ± 0.11*a	1.62 ± 0.03*	3.66 ± 0.36	3.37 ± 0.14	3.00 ± 0.06*	3.46 ± 0.21	3.35 ± 0.16	3.22 ± 0.08*
Serum (mg/dL)									
BUN	38.21 ± 0.16	52.67 ± 0.46*a	42.60 ± 0.38*	52.50 ± 0.62	53.26 ± 0.28	48.25 ± 0.63*	52.88 ± 0.32	54.20 ± 0.68	42.30 ± 0.12*
Creatinine	0.56 ± 0.03	0.98 ± 0.08*a	0.58 ± 0.01†	0.88 ± 0.20	0.84 ± 0.10	0.62 ± 0.03†	0.68 ± 0.11	0.64 ± 0.03	0.61± 0.06†
Uric acid	1.26 ± 0.04	3.48 ± 0.11*a	1.88 ± 0.03*	3.18 ± 0.07	3.32 ± 0.27	2.38 ± 0.09*	3.42 ± 0.28	3.28 ± 0.10	2.18 ± 0.01*

Data were analysed by ANOVA followed by Dunnett’s test. All values are expressed as mean ± SEM (n = 6);

*a Comparisions are made with Group I;

† *P* < 0.05.

†† *P* < 0.01.

* *P* < 0.001.

For *in vivo* antioxidant activity, ethylene glycol treatment increased MDA (*P* < 0.01) and decreased GSH (*P* < 0.05), SOD (*P* < 0.01), and CAT (*P* < 0.01) levels in control animals. AIE of *M. elengi* bark produced significant (*P* < 0.001) reduction in MDA and increased GSH and antioxidant enzymes like SOD and CAT compared to standard group cystone [Table 3].

**Table 3:** Effect of various extracts of *M. elengi* bark on antioxidant enzymes in renal tissue

*Treatment*	*Catalase activity (A/mg protein)*	*Superoxide dismutase activity (B/mg protein)*	*GSH (nmoles/mg protein)*	*MDA (nmoles/mg protein)*
Normal	3.13 ± 0.05	9.03 ± 0.15	3.64 ± 0.14	3.82 ± 0.26
Ethylene glycol	0.94 ± 0.03^a^	3.90 ± 0.15^a^	0.48 ± 0.15^a^	9.96 ± 0.54^a^
Cystone (750 mg/kg b.w.)	2.83 ± 0.18***	5.47 ± 0.17***	2.53 ± 0.18***	4.12 ± 0.32***
Petroleum ether extract (200 mg/kg b.w.)	1.12 ± 0.04ns	4.27 ± 0.16ns	0.94 ± 0.24ns	8.62 ± 0.36
Chloroform extract (200 mg/kg b.w.)	1.25 ± 0.11ns	3.81 ± 0.16ns	0.89 ± 0.18ns	8.47 ± 0.53ns
Alcohol extract (200 mg/kg b.w.)	2.46 ± 0.08***	6.08 ± 0.19***	1.87 ± 0.14***	5.10 ± 0.38***

Data were analysed by ANOVA followed by Dunnett’s test. Each value is the mean ± SEM; n = 6;

a *P* < 0.001 when compared to normal group;

*** *P* < 0.001 when compared to ethylene glycol treated group; A, Micro mole of H2O2 consumed per minute; B, one unit of activity was taken as the enzyme reaction, which gave 50% inhibition of NBT reduction in 1 min; MDA, malondialdehyde; GSH, glutathione.

## Discussion

Chronic administration of 0.75% ethylene glycol aqueous solution to male Wistar rats resulted in hyperoxaluria. Urinary stone formation is the result of supersaturation of urine with certain urinary salts such as calcium oxalate. Lower level of oxalate, calcium, and phosphate in urine and kidney reduces the risk of stone formation. AlE of *M. elengi* bark significantly lowered the levels of oxalate and calcium in urine and kidney.

Remarkable increase in urinary phosphate was observed in calculi-induced rats. Increased urinary phosphate excretion along with oxalate stress seems to provide an environment appropriate for stone formation by forming calcium phosphate crystals, which epitaxially induces calcium oxalate deposition. Treatment with AlE of *M. elengi* bark restores urinary phosphate level, thereby reducing the risk of stone formation. In urolithiasis, the glomerular filtration rate (GFR) decreases due to the obstruction to the outflow of urine by stones in urinary system. Due to this, the waste products such as urea, creatinine, and uric acid get accumulated in blood. In calculi-induced rats, the elevated serum levels of creatinine, uric acid, and BUN indicate marked renal damage.

This study also revealed the increased lipid peroxidation and decreased levels of antioxidant potential in the kidneys of rats supplemented with ethylene glycol. Oxalate, the major stone-forming constituent, has been reported to induce lipid peroxidation and cause tissue damage by reacting with polyunsaturated fatty acids in cell membranes. Phenolic compounds present in *M. elengi* may prevent the lipid peroxidation-induced renal damage caused by calcium oxalate crystal deposition in the kidney. Hence, *M. elengi* can prevent calcium oxalate crystal attachment as well as stones formation. *M. elengi* treatment produced significant decrease in MDA and increased GSH, SOD, and CAT. These results indicate the protective effect of AlE of *M. elengi* against the oxidative changes induced by ethylene glycol. These properties have been attributed to the triterpenes, lupeol,[19] and polyphenolic compounds like quercetin[20] present in *M. elengi*. Thus, the results reveal that the AlE of *M. elengi* possess a potent antiurolithiatic and antioxidant activity similar to pomegranate juice.[21]

Other possible mode of action includes excessive excretion or decrease in the concentration of urinary salts that prevent the supersaturation of the crystallizing salts. However, diuretic activity of *M. elengi* may hasten the process of dissolving the preformed stones in CR and prevention of new stone formation in urinary system on prophylactic treatment. The present investigation provides evidence for the efficacy of AlE of *M. elengi* bark in urolithiasis and confirms its utility in Folk medicine.

## References

[1] Khan SR (1997). Interactions between stone-forming calcific crystals and macromolecules. Urol Int.

[2] Alexander H, Nestor S (1999). *Phyllanthus niruri* inhibits calcium oxalate endocytosis by renal tubular cells: Its role in nephrolithiasis. Nephron.

[3] Freitas AM, Schor N, Boim MA (2002). The effect of *Phyllanthus niruri* on urinary inhibitors of calcium oxalate crystallization and other factors associated with renal stone formation. BJU Int.

[4] Grases F, March JG, Ramis M, Costa-Bauz´a A (1993). The influence of Zea mays on urinary risk factors for kidney stones in rats. Phytother Res.

[5] Grases F, Ramis M, Costa-Bauza A, March JG (1995). Effect of *Herniaria hirsuta* and *Agropyron repens* on calcium oxalate urolithiasis risk in rats. J Ethnopharmacol.

[6] Yasui T, Fujita K, Sato M, Sugimoto M, Iguchi M, Nomura S (1999). The effect of Takusya, a Kampou medicine, on renal stone formation and osteopontin expression in rat urolithiasis model. Urol Res.

[7] Dar A, Behbahanian S, Malik A, Jahan N (1999). Hypotensive effect of the Methanolic extract of *Mimusops elengi* in normotensive rats. Phytomed.

[8] Koti BC, Purnima A (2010). Diuretic activity of extracts of *Mimusops elengi* Linn. Bark. Int J Green Pharmacy.

[9] Atmani F, Slimani Y, Mimouni M, Hacht B (2003). Prophylaxis of calcium oxalate stones by *Herniaria hirsuta* on experimentally induced nephrolithiasis in rats. BJU Inter.

[10] Mitra SK, Gopumadhavan S, Venkataranganna MV, Sundaram R (1998). Effect of Cystone, a herbal formulation, on glycolic acid-induced urolithiasis. Phytotherapy Res.

[11] Medeiros DM, Mustafa MA (1985). Proximate composition, mineral content and fatty acids of cat fish (Ictalurus punctatus rafinesque) for different seasons and cooking methods. J Food Sci.

[12] Fiske CH, Subbarow Y (1925). The colorimetric determination of phosphate. J Biol Chem.

[13] Hodgkinson A, Williams A (1972). An improved colorimetric procedure for urine oxalate. Clinica Chimica Acta.

[14] Caraway WT, Seligson D (1963). Uric acid. Standard methods of clinical chemistry.

[15] Ellman GL (1959). Tissue sulfhydryl groups. Arch Biochem Biophys.

[16] Mishra HP, Fridovich I (1972). The role of superoxide anion in the auto-oxidation of Epinephrine and a simple assay for superoxide dismutase. J Biol Chem.

[17] Takahara S, Hamilton BH, Nell JV, Ogubra TY, Nishimura ET (1960). Hypocatalasemia: A new genetic carrier state. J Clin Invest.

[18] Ohkawa H, Ohish N, Yogi K (1979). Assay for lipid peroxidase in animal tissues by thiobarbituric acid. Anal. Biochem.

[19] Anand R, Patnaik GK, Kulshreshtha DK, Dhawan BN (1994). Antiurolithiatic activity of lupeol, the active constituent isolated from Crateva nurvala. Phytother Res.

[20] Park HK, Jeong BC, Sung M, Park M, Choi EY, Kim BS (2007). Reduction of oxidative stress in cultured renal tubular cells and preventive effects on renal stone formation by the bioflavonoid quercetin. J Urol.

[21] Tugcu V, Kemahli E, Ozbek E, Arinci YV, Uhri M, Erturkuner P (2008). Protective effect of a potent antioxidant, pomegranate juice, in the kidney of rats with nephrolithiasis induced by ethylene glycol. J Endourol.

